# Adult stem cell maintenance and tissue regeneration around the clock: do impaired stem cell clocks drive age-associated tissue degeneration?

**DOI:** 10.1007/s10522-018-9772-6

**Published:** 2018-10-29

**Authors:** Eve H. Rogers, John A. Hunt, Vanja Pekovic-Vaughan

**Affiliations:** 1 Institute of Ageing and Chronic Disease, University of Liverpool, The William Henry Duncan Building, 6 West Derby Street, Liverpool, L7 8TX UK; 2 School of Science and Technology, Nottingham Trent University, Clifton Campus, College Drive, Nottingham, NG11 8NS UK

**Keywords:** Adult stem cells, Circadian rhythms, Aging, Proliferation, Differentiation, Mechanical stimulation

## Abstract

Human adult stem cell research is a highly prolific area in modern tissue engineering as these cells have significant potential to provide future cellular therapies for the world’s increasingly aged population. Cellular therapies require a smart biomaterial to deliver and localise the cell population; protecting and guiding the stem cells toward predetermined lineage-specific pathways. The cells, in turn, can provide protection to biomaterials and increase its longevity. The right combination of stem cells and biomaterials can significantly increase the therapeutic efficacy. Adult stem cells are utilised to target many changes that negatively impact tissue functions with age. Understanding the underlying mechanisms that lead to changes brought about by the ageing process is imperative as ageing leads to many detrimental effects on stem cell activation, maintenance and differentiation. The circadian clock is an evolutionarily conserved timing mechanism that coordinates physiology, metabolism and behavior with the 24 h solar day to provide temporal tissue homeostasis with the external environment. Circadian rhythms deteriorate with age at both the behavioural and molecular levels, leading to age-associated changes in downstream rhythmic tissue physiology in humans and rodent models. In this review, we highlight recent advances in our knowledge of the role of circadian clocks in adult stem cell maintenance, driven by both cell-autonomous and tissue-specific factors, and the mechanisms by which they co-opt various cellular signaling pathways to impose temporal control on stem cell function. Future research investigating pharmacological and lifestyle interventions by which circadian rhythms within adult stem niches can be manipulated will provide avenues for temporally guided cellular therapies and smart biomaterials to ameliorate age-related tissue deterioration and reduce the burden of chronic disease.

## An introduction to adult stem cells

Pioneering experiments in stem cell research are often credited to Canadian scientists Till and McCulloch in 1961–1963, who were the first to carry out clonal colony formation assays on hematopoietic stem cells (HSCs) in murine bone marrow, indicating their multipotency (Becker et al. 1963). Since then, stem cell research has progressed exponentially, with numerous scientific breakthroughs. In this review, we will introduce some of the well-studied as well as emerging mechanisms regulating adult stem cell function and how these become affected during ageing. Recently, there has been an increased interest in several research areas pertaining to stem cell function in 3D environments including the role circadian clocks play in stem cell physiology (temporal regulation) as well as the role of mechano-transduction processes in modulating stem cell function (spatial regulation). This review aims to bring together developments in these exciting research fields and reveal important areas for novel research to be conducted. Further insights into these spatio-temporal mechanisms will allow better understanding of environmental cues that regulate stem cell physiology in vivo and aid future design of new stem cell therapies for age-related diseases.

Stem cells are characterized by their extraordinary capacity to both self-renew through cell divisions and to differentiate into a wide range of tissue-specific cells in response to endogenous or external stimuli. It is these regenerative roles that stem cells within each tissue niche carry out to repair tissues following injury/disease and maintain tissues throughout lifecourse. Indeed, adult stem cells (ASCs), which can be more appropriately referred to as adult progenitor cells, are being used to treat an ever-increasing array of human conditions, varying from heart disease to leukaemia. But in order for ASCs to become a therapeutic reality, these tissue regenerative processes must fully be understood, and the environmental factors that regulate this regeneration process fully uncovered, which is vital for the repair and replenishment of most tissues in the body.

Adult stem cell (ASC) classification has become highly complex since the original terms ‘haematopoietic’ and ‘mesenchymal’ covered the main types of these pluripotent cells. Currently, they tend to be classified based on their tissue of origin and differentiation potential, and one should perhaps increasingly replace the use of the term adult ‘stem cells’ in favour of adult ‘progenitor cells’. Adult progenitor cells, being somatic stem cells, can be derived from all parts of the body, where they are found in tissue-specific stem cell niches. Progenitor cells have been derived and utilised from cord and peripheral blood, blood vessels and bone marrow (mesenchymal and hematopoietic stem cells), the brain (neural stem cells), skin (epidermal stem cells), skeletal muscle (muscle satellite cells/myogenic stem cells), teeth (dental pulp stem cells), heart (cardiac stem cells), gut (intestinal stem cells), liver (hepatic stem cells), ovarian epithelium (ovarian stem cells), breast (mammary stem cells) and testis (testicular stem cells).

**Fig. 1:**
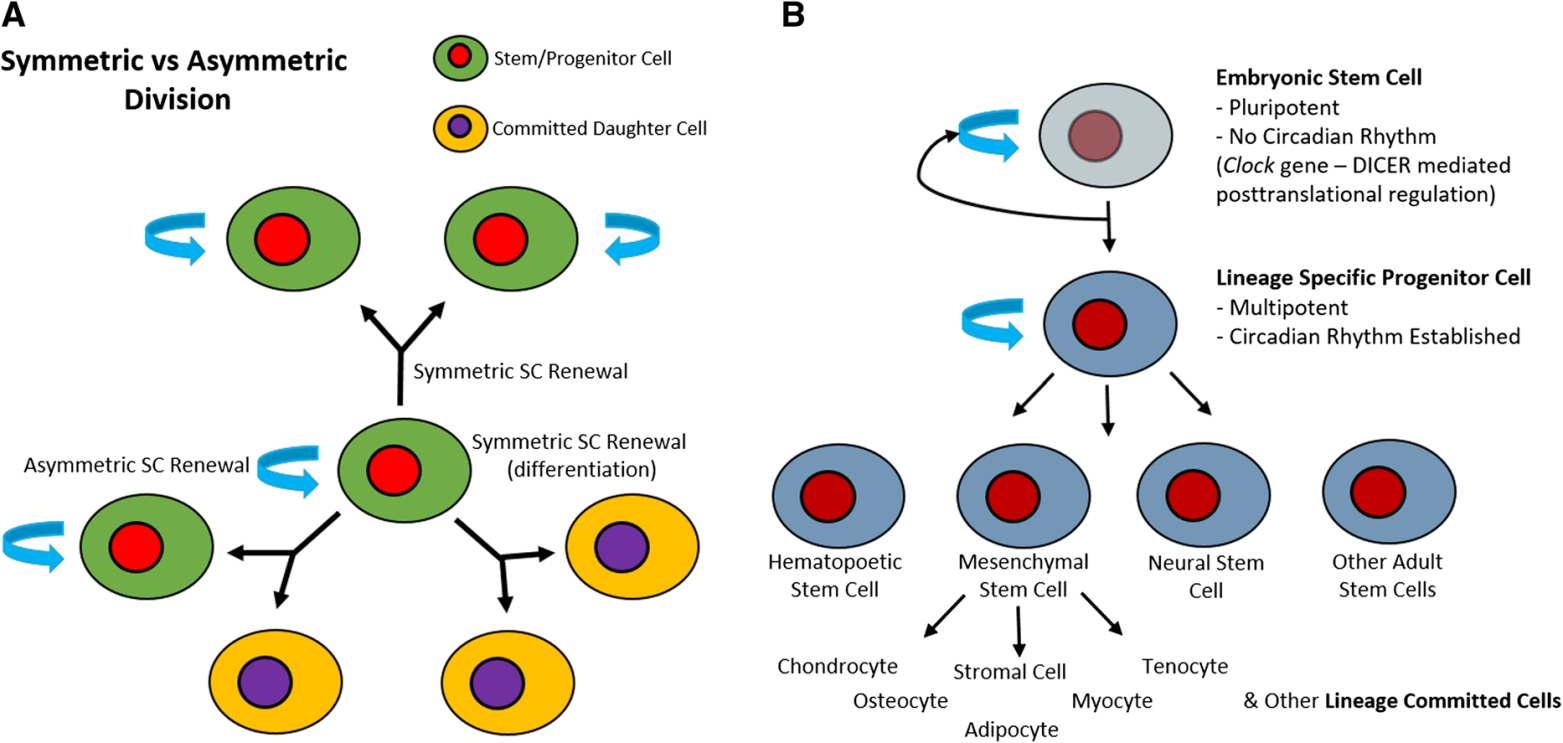
Stem Cell Division. **a** Adult stem cells are capable of dividing either symmetrically, to produce two identical stem cells or two identical daughter cells, or asymmetrically, to produce one identical stem cell and one committed daughter cell. **b** The hierarchy of stem cell division

ASCs are multipotent and are, by that definition, limited to giving rise to different cell types from their tissue of origin. They divide either symmetrically to produce two identical cells which self-renew, proliferate and expand in number, or asymmetrically to produce one identical stem cell and one committed daughter cell which maintain progeny population (Fig. 1). The form of division that occurs depends on developmental and environmental signals. It has been suggested that most ASCs have the ability to switch between asymmetric and symmetric division models, and that the balance between the two is often altered in disease states (Morrison and Kimble 2006).

When comparing embryonic stem cells to ASCs, embryonic stem cells (ESCs) are pluripotent, meaning that they are capable of differentiating into any one of the three germ layers; endoderm, mesoderm or ectoderm. Since their discovery, there has been a great interest in their use in regenerative medicine and tissue engineering, due to their pluripotent differentiation capabilities. However, the pluripotency of ESCs makes it difficult to direct their differentiation in a reliable, long term and reproducible manner. Furthermore, many in vivo studies have shown embryonic stem cells, following implantation, can spontaneously differentiate and form a type of tumour called a teratoma (Thomson et al. 1998). ASCs do not demonstrate these limitations in in vivo models, and so there is a substantial interest in using ASCs in regenerative medicine. However, ASCs are more committed than ESCs, and so they have a more limited differentiation potential. Nevertheless, ASCs such as bone marrow derived mesenchymal stem cells (BM-MSCs), hematopoietic stem cells (HSCs) from bone marrow and cord blood, and adipose-derived stem cells (ADSCs) are all attractive targets as they still have varied differentiation potentials and are able to differentiate into a variety of cell types. For example, BM-MSCs are able to differentiate into bone, cartilage, fat, tendon, muscle, and marrow stroma (Pittenger et al. 1999), and HSCs are able to differentiate into both myeloid (including monocytes, macrophages, neutrophils, basophils, eosinophils, erythrocytes, dendritic cells, and megakaryocytes or platelets) and lymphoid (T cells, B cells, and natural killer cells) lineages of blood cells. These ASCs are also advantageous as they present an ease of harvest, isolation and expansion in vitro, when compared to embryonic stem cells. The impressive multi-lineage differentiation potential of ASCs is made possible by the broad combination of chemical, biological and physical signals present in their stem cell niches, which direct and control their fate.

## A brief introduction to the body’s pacemaker: the circadian clock

An important evolutionarily conserved mechanism that becomes altered with age is the circadian clock, the body’s innate time-keeping system. Circadian rhythms are a subset of biological rhythms, which have a period of approximately 24 h. The foundation of circadian rhythmicity research is often dated back to the work done by Colin Pittendrigh and Jurgen Aschoff. These pioneers have defined the basis of circadian entrainment. Pittendrigh (1960) showed that deviation from the 24 h cycle provides a mechanism for alignment for the internal time-keeping system, allowing the rhythm to be “reset” where necessary (Pittendrigh 1960). In line with this anticipatory daily role of the circadian clocks, there are a number of mammals living in extreme conditions without a functioning circadian clock, which can be restored upon appropriate environmental stimuli. For example, indigenous Arctic reindeer do not express circadian rhythms during the periods of constant sunlight in the summer or constant darkness in winter months in the Arctic (Lu et al. 2010). Similarly, free-living Arctic ground squirrels do not express circadian rhythms during hibernation in the winter but do exhibit robust daily circadian body temperature oscillations over 24 h during their active season (Williams et al. 2011, 2017). In this way, such mammals are able to restablish circadian rhythmicity coincident with emergence to the surface and the resumption of surface activity (Williams et al. 2012).

**Fig. 2:**
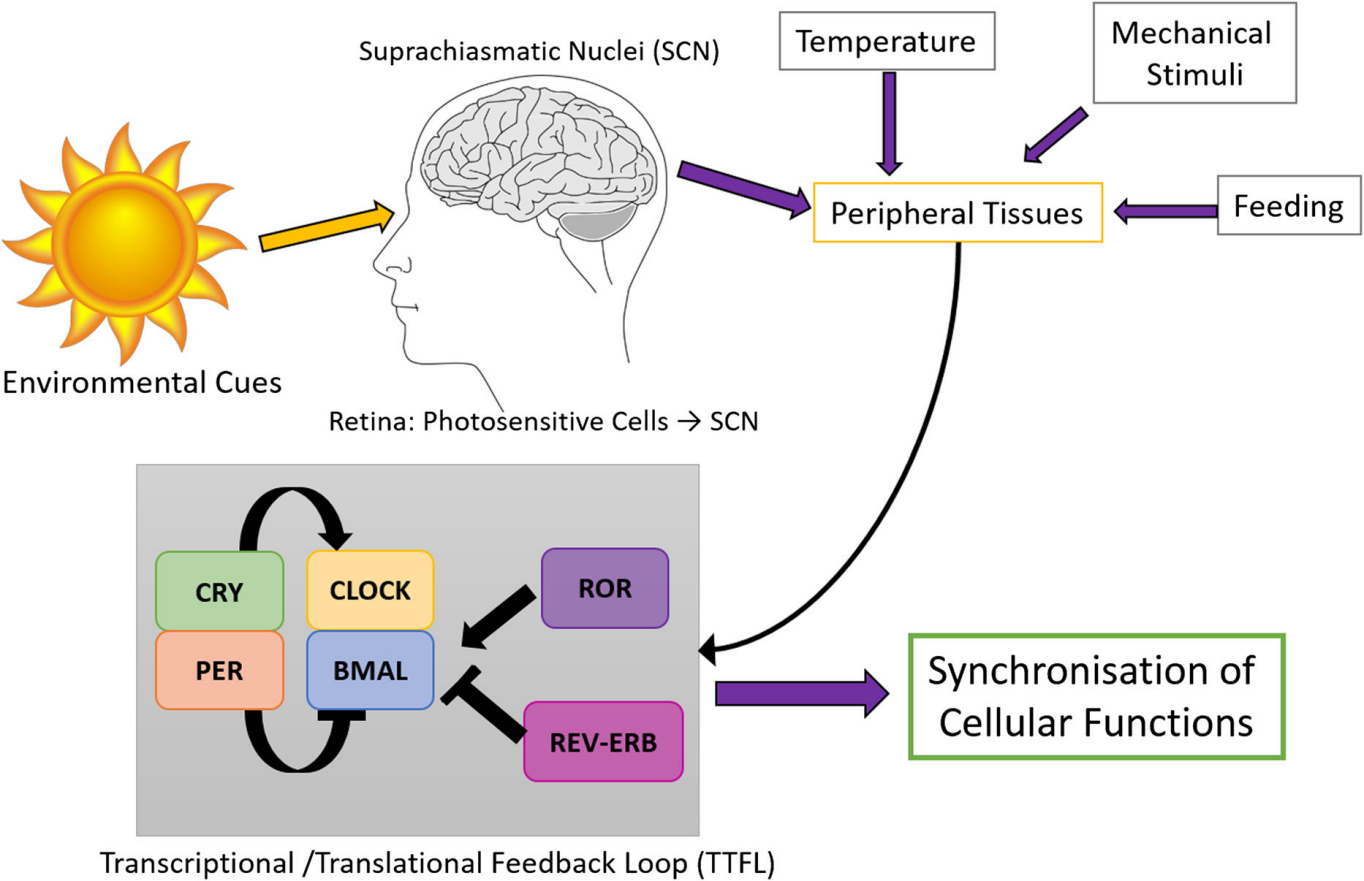
The circadian system organisation and the molecular clock. Light enters the retina via photoreceptor cells and is transduced to the ‘master pacemaker’, the suprachiasmatic nucleus (SCN), in the anterior hypothalamus of the brain. The SCN relays signals to the ‘peripheral tissue clocks’ throughout the body. At a molecular level, circadian clocks are regulated via primary and stabilising feedback loops, which regulate core clock genes within each loop and numerous target genes in each tissue (clock-controlled genes, CCGs), leading to synchronisation of tissue-specific cellular functions (modified from Rogers et al. (2018) with persmission from BioMedicine)

The mammalian circadian rhythms are orchestrated by a hierarchy of oscillators. The master clock located within the suprachiasmatic nucleus (SCN) in the anterior hypothalamus of the brain, coordinates a number of independent central nervous system (CNS) and peripheral tissue oscillators, acting as a pacemaker to regulate a coherent rhythm at the level of the whole organism (Yamazaki et al. 2000). The input pathway for the master pacemaker encompasses the light information that enters the retina through retinal photoreceptors, which is relayed to and entrains the SCN, which, in turn, sends neuro-endocrine signals that result in synchronisation of peripheral tissue clocks. The molecular mechanism that generates cell-autonomous, self-sustained circadian rhythms is governed by a network of auto-regulatory feedback loops of transcription/translation that drive circadian expression patterns of genes, proteins and metabolites within each tissue (Reppert and Weaver 2002). In mammals, this is carried out by the primary feedback loop which includes the basic-helix-loop-helix transcription factors CLOCK (Circadian Locomotor Output Cycles Kaput) and BMAL (also known as ARNTL, Aryl hydrocarbon Receptor Nuclear Translocator-Like protein 1), which form the positive arm of the molecular clock. When these two proteins heterodimerise, they are able to bind to *cis*-regulatory enhancer sequences called E-boxes on target gene promoters, and initiate transcription of numerous genes (King et al. 1997; Gekakis et al. 1998). Target genes include core clock components such as *Period* (*Per1, Per2* and *Per3*) and *Cryptochrome* (*Cry1* and *Cry2*) as well as numerous clock-controlled genes (CCGs). PER and CRY proteins then heterodimerise and translocate to the nucleus. They form a negative arm of the feedback loop, and repress target gene expression, including their own transcription, by inhibiting the CLOCK:BMAL complex (Kume et al. 1999; Shearman et al. 2000). The CLOCK:BMAL heterodimer also induces a stabilising regulatory loop by activating the transcription of Retinoic Acid-Related (RAR) orphan nuclear receptors, *Rev*-*erb* (also known as *Nr1d1, Nuclear Receptor subfamily 1, group D, member 1*) and *Ror* (also known as *Nr1f1, Nuclear Receptor subfamily 1, group F, member 1*). These bind to retinoic acid-related orphan receptor response elements (ROREs), which are present in many clock-controlled gene promoters as well as the core clock gene *Bmal*. REV-ERB represses transcription of *Bmal*, whereas ROR activates it (Guillaumond et al. 2005). These auto-regulatory loops constitute a molecular clock machinery and take approximately 24 h to complete a cycle. A diagram depicting the circadian system organisation in mammals and the components of the molecular clock are depicted in Fig. 2.

There has been a number of recent exciting studies documenting how ageing affects the molecular clock in several tissues (Kondratov et al. 2006). Mouse models of genetic clock disruption show premature ageing in many tissues. For example, circadian disruption of *Bmal1* led to muscle loss and sarcopenia (Andrews et al. 2010), disrupted cartilage formation (Dudek et al. 2016), bone loss and other features of premature ageing (Kondratov et al. 2006). This showed that genetic disruption of the circadian clock not only leads to circadian arrhythmia, but also degenerative changes in many tissues that are associated with advanced age. Future work will reveal how much of tissue degeneration resulting from *Bmal1* deficiency is caused by impaired *Bmal1*-dependent stem cell homeostasis, which may be responsible for some of the age-related changes seen in various tissue systems.

The link between circadian disruption and adult stem cell function during ageing has been investigated recently by comparing the clock dynamics in epidermal and muscle stem cells isolated from adult and old mice (Solanas et al. 2017). Unexpectedly, the authors demonstrated that adult stem cells from aged mice (≥ 18 months) retained circadian rhythms and that the core clock machinery of the epidermal and muscle stem cells remained robustly rhythmic. However, it was shown that there was an extensive reprogramming of the oscillating transcriptome in the aged stem cells, switching from homeostatic genes to genes involved in tissue-specific stresses, for example DNA damage or autophagy. It was concluded that physiological tissue ageing was associated with the rewiring of stem cell’s diurnally timed functions, rather than with the arrhythmia. The former is hypothesized to be switched on in older organisms in order to adapt to metabolic cues and tissue-specific age-related traits. This age-associated rewiring of the oscillatory diurnal transcriptome was significantly rescued in old mice by long-term caloric restriction (CR), known to reverse the effects of ageing. As CR diet is known to uncouple the cycling of peripheral tissues from the SCN, this rewiring may be driven by the molecular changes in the SCN.

The role of circadian clock genes in stem cell function has also been investigated in *Clock*^Δ19^ mutant mouse model with inactive BMAL1/CLOCK transcriptional complex (Yang et al. 2017). It was shown that mammospheres arising from mammary adult stem cell progenitors demonstrated rhythmic PER2::luc oscillations, which were dampened in the mutant mice. Importantly, the ability of the *Clock*^Δ19^ cells to form mammospheres was considerably reduced, showing that mice containing genetically disrupted clocks have impaired stem cell function and renewal. This further supports the hypothesis that the age-associated changes seen in mouse models with genetically disrupted clocks may be driven by the clock dysfunction within tissue-specific stem cells.

## The implications of ageing on stem cell self-renewal, proliferation and differentiation

One physiological process that has been implicated in the loss of stem cell homeostasis with age, and is under the control of circadian rhythms, relates to tissue regeneration. Despite the promising advances made using ASCs in tissue engineering and regenerative medicine, challenges remain due to diminished tissue regeneration seen with ageing when either utilising stem cells from old donors or treating an older patient. Upon ageing, ASCs exhibit reduced proliferation and differentiation which are thought to contribute to tissue deterioration, due to a decline in cell function and/or decreased numbers with age (Boyle et al. 2007). As ASCs have key role in the maintenance of several organs and tissues, any decrease in their number and/or function can seriously compromise the maintenance of tissues and contribute to a number of age-related phenotypes. For example, studies have shown that cell-extrinsic changes contribute to a decline in stem cell abilities to repair damaged tissues with age in muscle progenitor cells (Conboy et al. 2005).

The age-associated phenotype in ASCs has predominantly been studied in human mesenchymal stem cells (MSCs). Morphologically, old MSCs appear larger and more flattened when they reach approximately 40 population doublings and stop proliferating (Bruder et al. 1997). These age-related changes are associated with cellular senescence in vitro. The cells do not only appear larger in size, but show increased spreading and display more podia (Mauney et al. 2004), as well as produce more actin stress fibres (Stenderup et al. 2001). Furthermore, with each passage, the MSCs experience a shortening of their telomeres. As MSCs from younger donors have longer telomeres, they can expand over many population doublings in vitro, proliferate faster and express pluripotency markers such as *Oct-4*, *Nanog*, *Rex-1*, *SSEA-3*, *SSEA-4*, *Tra-1-60*, and *Tra-1-81* (Guillot et al. 2007). Furthermore, chronological age has been shown to influence the proliferation rate of ASCs in rodents (Fafián-Labora et al. 2015). MSCs isolated from older donors vary in their expression of proliferation marker Ki67, with the reduction in Ki67 corresponding to lower proliferation rates whilst increases seen in self-renewal marker CD117 correspond to higher cell numbers.

Moreover, ASCs harvested from older donors show that the frequency of MSCs in bone marrow is significantly lower than in young donors (Tokalov et al. 2007). Using methods such as flow cytometry to determine the proportions of cells from different cell lineages within bone marrow isolated from rats of different ages, it has been demonstrated that bone marrow consists of three main populations of nucleated cells; polynuclear cells (PNCs), megakaryocytic cells (MKCs) and mononuclear cells (MNCs), and the proportions of these populations differs with age. During ageing, an increase in PNCs, a decrease in MNCs and a limited change in the relative number of MKCs was observed. Within the CD90 + MNC population, the number of MSCs significantly decreased with age due to a decrease in the maximal lifespan of these cells.

Upon appropriate stimulation, MSCs give rise to a number of different mesenchymal cell types, most frequently undergoing osteogenesis, adipogenesis, chondrogenesis or myogenesis. These distinct cellular fates are defined by their particular patterns of gene expression. When MSCs differentiate, they switch from one pattern of gene expression to another; the lineage is determined by the activation of phenotype-specific transcription factors, such as the adipocyte specific PPAR-γ2 (Tontonoz et al. 1994) or the osteoblast specific RUNX2/CBFA-1 (Ducy et al. 1997). Interestingly, it has been shown that despite increased markers of senescence in MSCs isolated from older animals, aged MSCs and ADSCs retain their differentiation potential into particular cell fates such as into Schwann cells (Mantovani et al. 2012). Similarly, it was documented that the endothelial differentiation potential of MSCs does not change with age. However, research by Fafián-Labora et al. (2015) showed, in contrast, that MSCs isolated from older rats exhibited a significantly lower differentiation potential than those from younger rats, when induced to differentiate into the osteogenic, chrondrogenic or adipogenic cell fates (Fafián-Labora et al. 2015). The authors also reported that the MSCs isolated from the older group of rats exhibited significantly lower amounts of *Nanog*, implicating that MSCs from older donors have a lower pluripotency potential. It was also demonstrated that the metabolic profiles significantly differed between these different age groups. The authors carried out iTRAQ analysis to compare global profile of proteins from MSCs of rat bone marrow at different ages and discovered increases in metabolic proteins including lactate dehydrogenase in MSCs from adult rats, which suggested increased rates of glycolysis. This was further supported by differences observed in the pentose phosphate pathway activity, whereby decreases in glucose 6 phosphate dehydrogenase activity were seen in the pre-pubertal group whilst increases were observed in the adult group.

Furthermore, a study by Han et al. (2012) sought to ameliorate these age-related changes seen in MSCs to improve decreased proliferative capacity and myogenic differentiation potential with age (Han et al. 2012). To combat this, the authors ectopically expressed the pluripotency marker *Nanog* using lentiviral transduction in BM-MSCs from neonatal and adult donors. They discovered that *Nanog* re-expression did indeed ameliorate reductions in proliferation and myogenic differentiation with age. Several signalling pathways that mediate these changes were identified, including the PPAR signalling pathway which was significantly altered in BM-MSCs upon *Nanog* expression, with both adipogenic genes *CEBPα* and *PPARγ* becoming downregulated. The differentiation of BM-MSCs into smooth muscle cells was also enhanced by *Nanog* expression, as demonstrated by increased contractility, myogenic function and an increased expression of smooth muscle cell markers such as smoothelin, SM22 and caldesmon (Han et al. 2012). This research suggested that the ectopic expression of *Nanog* may rescue age-mediated decline in BM-MSC functions, which could allow for the use of BM-MSCs from older donors in regenerative medicine.

Osteogenic progenitors also show a reduced capacity for self-renewal in vivo with age (Bellows et al. 2003). Indeed, there is an increased number of adipocytes in the old bone marrow and a decreased number of bone-forming osteoblasts, accompanied by reductions in bone mass. This reduction in osteogenic potential and acquired adipogenic potential with age is referred to as an ‘adipogenic switch’ (Ross et al. 2000). Research carried out by Moerman et al. (2004) showed that bone marrow aspirates of old mice gave rise to fewer osteoblastic colonies and more adipocytic colonies, when compared to adult mice. From this, they concluded that ageing alters the differentiation potential of MSCs, leading to cells being more likely adipogenic than osteogenic. Using a PPAR-γ2 agonist, rosiglitazone, the authors demonstrated that sensitivity to PPAR-γ is increased in the old bone marrow, and that TGF-β/BMP signalling pathways are altered with age (Moerman et al. 2004). In addition, Gharibi et al. (2014) sought to ameliorate the reductions in osteogenesis seen in vitro in MSCs with age (Gharibi et al. 2014). To do this, the authors demonstrated that blocking AKT/mTOR pathway prevented the development of this age-related phenotype, maintained the MSC morphology and enhanced proliferation capacity, matching those seen in early passage MSCs. MSCs cultured with inhibitors of AKT and/or mTOR also maintained their osteogenic potential. It was speculated that these effects may be influenced by the expression of pluripotency genes such as *Nanog* and *Oct*-*4* and due to a reduction in reactive oxygen species production (Gharibi et al. 2014). Other research has shown that growing BM-MSCs on certain biomaterials preserved age-compromised functions, maintained the differentiation potential and enhanced proliferation capacity. BM-MSCs were grown on denatured collagen matrices which significantly influenced the protective stress responses and proliferation capacity ex vivo, reduced the rate of morphological alterations and increased the osteogenic potential of old BM-MSCs (Mantovani et al. 2012). Overall, understanding the mechanisms that underlie the ageing processes within ASCs is vital both in terms of ameliorating the age-related tissue deterioration, but also for selecting the appropriate biological age of stem cell donors and designing future stem cell therapies for older patients.

## Circadian clocks as a novel mechanism for temporal control of stem cell function

A recent field of investigation has shown that the clock genes can directly influence adult stem cell activation and differentiation, within their tissue-specific niches (Brown 2014; Aguilar-Arnal and Sassone-Corsi 2011; Plikus et al. 2015). The core circadian clock genes, *Per1, Per2, Bmal1, Cry1, Clock,* and *Rev*-*erbα*, were first characterised as actively cycling in mouse HSCs using a combination of cell sorting by high-speed flow cytometry and gene expression analyses (Tsinkalovsky et al. 2005). The circadian clock genes are well documented to be expressed in an oscillatory manner in murine adipose tissue, following which they were also investigated in human subcutaneous adipose-derived stem cells (Wu et al. 2007). Here, cells were synchronised in vitro with synthetic glucocorticoid dexamethasone, PPAR- γ2 agonist rosiglitazone, or 30% foetal bovine serum, and total RNA collected every 4 h over a 48 h period. The authors reported that differentiated adipocytes were more readily responsive to clock synchronisation than undifferentiated pre-adipocyte precursors, but the period of clock gene oscillations were longer in differentiated adipocytes, validating the use of ADSCs as in in vitro adult stem cell model for the analysis of circadian rhythms (Wu et al. 2007). In MSCs, Wu et al. (2008) reported the presence of the core circadian transcriptional machinery in both murine and human primary BM-MSCs, and when exposed to the synchronising effects of the synthetic glucocorticoid, dexamethasone, BM-MSCs showed oscillating expression of the mRNAs encoding *Bmal1, Per3, Albumin D Binding Protein (Dbp), Rev*-*erbα* and *Rev*-*erbβ* (Huang et al. 2009). Furthermore, circadian oscillations were also elicited when BM-MSCs and ADSCs were exposed to serum shock and cAMP analogs (Huang et al. 2009). The circadian mechanisms that have been recently used to synchronise adult stem cells in vitro can be found in Table 1, along with rhythmic temperature cycles, which have been used to synchronise human red blood cells (RBCs) (O’Neill and Reddy 2011).

**Table 1:** The Circadian Synchronisation Mechanisms investigated in Adult Stem Cells

Entrainment mechanism	References	Adult stem cell type
cAMP agonists (forskolin)	Huang et al. (2009)	BM-MSCs, ADSCs
Glucocorticoids (dexamethazone)	Wu et al. (2007, 2008)	BM-MSCs
Growth factors (serum shock)	Huang et al. (2009)	BM-MSCs, ADSCss
Temperature	O’Neill and Reddy (2011)	RBCs
Mechanical stretch	Simoni et al. (2014) Rogers et al. (2017)	*Drosophila Melanogaster* BM-MSCs, ADSCs, DPSCs
Biomaterials	Mengatto et al. (2011) Hassan et al. (2017)	Mice BM-MSCs

There has been a substantial amount of evidence that suggests that not only do ASCs express the functional core circadian machinery, but that circadian clocks have important role in a variety of tissue homeostatic functions thus influencing the activation of stem and progenitor cells (Gimble et al. 2009; Weger et al. 2017). Circulating HSCs and their progenitors in the bloodstream have been shown to exhibit robust circadian oscillations in light/dark entrained animals (Méndez-Ferrer et al. 2008). Here, it has been experimentally evidenced that the release of HSCs into the bloodstream is cyclical, along with the rhythmic expression of *C-X-C motif chemokine 12 (Cxcl12)*, both of which are regulated by the core molecular clock through rhythmic noradrenaline secretion. This implies that the clock-driven release of HSCs is stimulated by the CNS during the animal’s resting phase and can promote the tissue regeneration and regulate the function of a hematopoietic stem cell niche.

Consequently, any disturbances in this temporal coordination have been implicated in a variety of pathologies including premature ageing and cancer. Indeed, recent studies have implicated circadian rhythm disruption in an increased susceptibility to cancer development in all major organ systems (Fu and Kettner 2013). Research by Puram et al. (2016) utilised a series of in vivo RNA interference screens to identify which transcription factors influenced a murine model of acute myeloid leukaemia (AML) (Puram et al. 2016). It was demonstrated that *Clock* and *Bmal1* were required for the growth of AML cells both in vitro and in vivo, and that the disruption of canonical circadian components led to anti-leukemic effects. RNA interference screens were also used to examine the effects of DNA damage and ageing on the maintenance of HSCs (Wang et al. 2016). It was discovered that *Per2* is activated in lymphoid-biased HSCs (as opposed to myeloid-biased HSCs) and, in hematopoietic cells, wherein it stimulates DNA damage responses and p53-dependent apoptosis. Therefore, *Per2* has been identified as a negative regulator of lymphoid-biased HSCs, immune function and lymphopoiesis. Another core clock gene that has been implied in cancer stem cells is *Per3*. *Per3* has been shown to have an important role in colorectal cancer, being downregulated in colorectal cancer stem cells (Zhang et al. 2017). As *Per3* has been reported to have a critical role in colorectal cancer stem cells pluripotency, it may be a promising gene for targeting cancer stem cells. Furthermore, circadian rhythms have been linked to tumour cell proliferation by regulating iron metabolism; iron is essential for DNA synthesis and therefore critical in the enhanced rates of proliferation seen in tumours (Okazaki et al. 2016). Circadian variations in DNA synthesis and proliferation are seen in tumour cells, and 24-h rhythms can be observed in iron regulatory protein 2 (IRP2). IRP2 regulates the 24 h rhythm in *transferrin receptor 1* mRNA post-transcriptionally, and *Irp2* is promoted by BMAL1:CLOCK heterodimers, demonstrating a role for the circadian clock in tumour cell stem proliferation by regulating iron metabolism.

## The circadian rhythm regulates adult stem cell activity at a multi-cellular level

Recent data has demonstrated that the involvement of circadian clocks in the regulation of adult stem cell activity is not only cell-specific, but, remarkably, can also act at the cell population level. In a study by Janich et al. (2011), it was shown that the circadian clock may have a role in regulating the activation of coexisting epidermal stem cell populations, which are in different phases, in order to balance hair growth and renewal (Janich et al. 2011). The authors noted that the genes regulating niche dormancy, activation and differentiation contained several putative BMAL1/CLOCK-binding sites, as revealed by gene promoter analysis. These key epidermal homeostasis genes included WNT signalling factors, TGF-β regulators and modulators of BMP and NOTCH signalling pathways. Chromatin immunoprecipitation (ChIP) assays confirmed the binding of BMAL1/CLOCK to these gene promoters in adult tail epidermis, and that the binding of BMAL1 to these target gene promoters was rhythmic. Therefore, the molecular clock generates cell populations that show heterogeneous responses to external factors, by modulating the expression of stem cell regulatory genes in an oscillatory manner. Furthermore, the deletion of *Bmal1* led to circadian arrhythmia, decreased expression of WNT-related genes and TGF-β inhibitors, and caused a progressive accumulation of dormant stem cells and premature epidermal ageing. In contrast, deleting the negative clock loop components *Per1/2*, resulted in progressive depletion of dormant stem cells, which may have implications for cancer.

A subsequent study using human keratinocyte progenitors showed that these cells responded better to several differentiation cues at certain times of the day (Janich et al. 2013). Interestingly, genes encoding key proliferation or differentiation proteins were expressed at different times of the day. For example, DNA replication and cell division pathways were highly represented in the evening, whilst differentiation pathways predominated in the morning. Moreover, the circadian clock coordinated the activities of glycolysis and oxidative phosphorylation with DNA synthesis in proliferating stem cells, most likely as a protective mechanism to prevent genotoxicity. Disruption of this clock-controlled mechanism in stem cells may therefore contribute to stem cell dysfunction and have long-term consequences for tissue homeostasis. Further work in hair follicle cycling has shown that prominent daily mitotic rhythms are generated by peripheral circadian clock within epithelial matrix cells (Plikus et al. 2013), which results in the hair growing faster in the morning than evening and therefore being exposed to higher exposure to genotoxic stress at certain times of the day. Researchers exposed wild-type mice to γ-radiation in the morning (mitotic peak) versus the evening (when there is minimal hair loss), and reported that the diurnal radio-protective effect is lost in clock mutant mice. The circadian clock was demonstrated to coordinate genotoxic stress responses with cell cycle progression by influencing the Cdc2/Cyclin B-mediated G2/M checkpoint. Further research demonstrating the links between various mammalian peripheral tissue clocks and downstream effects on tissue-specific functions, including liver, pancreas, adipose, skeletal muscle, brain, intestine, hematopoietic and immune system, skin and cartilage, has been reviewed in detail (Janich et al. 2014).

In contrast to ASCs, the circadian transcriptional machinery does not seem to oscillate in ESCs, when analysed using real-time bioluminescent imaging systems (Yagita et al. 2010). However, upon differentiation in vitro, a molecular clock oscillations become strongly induced, which can be reversed if the differentiated cells are reprogrammed into induced pluripotent stem cells (IPSCs) using the four pluripotency factors *Oct3/4*, *Sox2*, *Klf4*, and *c*-*Myc* (Paulose et al. 2012). This suggests that formation of the circadian molecular oscillator is dependent on an intrinsic program that occurs during cellular stem cell differentiation. When ESCs are maintained in a pluripotent state in culture, it has been discovered that they express a self-sustained rhythm in glucose uptake that is not coincident with clock gene oscillations, and this rhythm is paralleled by rhythmic glucose transporter mRNA expression, indicating that circadian rhythms in metabolism emerge earlier than clock gene expression rhythms (Paulose et al. 2012). When stem cells become differentiated, however, circadian patterns in clock gene expression can be observed, and the glucose utilization rhythm becomes enhanced in amplitude, providing the evidence of a circadian clock in differentiated stem cells. Further experiments carried out by Lu et al. (2016) have demonstrated that when *Clock* is knocked out entirely using CRISPR/CAS9-mediated genetic editing techniques in mouse ESCs, there was no influence on the cellular pluripotent state, but mESCs did exhibit a decreased proliferation rate and an increased apoptosis. Interestingly, clock gene rhythms failed to develop in these mESCs following differentiation, suggesting that *Clock* may be critical in mESC differentiation (Lu et al. 2016). These findings have been supported by research in mouse embryonic hearts and ESCs which demonstrated a role for the posttranscriptional regulation of *Clock* in development of molecular clock oscillations in differentiating stem cells. Indeed, the appearance of CLOCK protein during ESC differentiation coincides with the emergence of molecular clock oscillations and *Dicer/Dgcr8*-mediated posttranscriptional regulation of CLOCK protein (Umemura et al. 2017), highlighting the importance of CLOCK in the establishment of circadian clock oscillations during embryonic stem cell differentiation.

Mechanistically, it has been shown that expressing both transcription factor *c*-*Myc* and ablation of DNA methyltransferase 1 (*Dnmt1*) leads to disruption in the establishment of the molecular clock oscillations in differentiating mouse ESCs. It has been reported that, when there is a failure of clock oscillation development, an increase in *Kpna2* (*Importin*-*α2*) and altered subcellular PERIOD protein localization can be observed; therefore differentiation-coupled transcription of specific genes may regulate circadian clock development in mouse ESCs (Umemura et al. 2014). Furthermore, ESCs have immature mitochondria, are reliant on glycolysis for fuel and have no discernible rhythms, compared to differentiated stem cells which have mature mitochondria, acquire oxidative respiration and show clear rhythms, linking redox regulation to stem cell differentiation and the development of circadian clocks. Indeed, clear redox oscillations can be imaged in proliferating epidermal stem cells, demonstrating the role for circadian clocks in regulating metabolism in adult stem cells (Stringari et al. 2015). The adult stem cell clock coordinates the activities of DNA synthesis with glycolysis and oxidative phosphorylation, whereby increased glycolysis is found during the night, along with a higher proportion of stem cells in S phase (when DNA synthesis occurs) (Stringari et al. 2015). This temporal segregation of metabolic processes from stem cell proliferation is thought to act as a protective mechanism against genotoxicity.

## The molecular clock exerts essential regulation of stem cell differentiation fate

### Adipogenesis

In addition to their role in stem cell activation, circadian rhythms have also been extensively linked to stem cell proliferation and differentiation. It is well established that *Bmal1* is involved in the regulation of adipogenesis and lipid metabolism in mature adipocytes. When 3T3-L1 cells are subjected to adipogenic differentiation, the level of *Bmal1* mRNA increases and it is highly expressed in differentiated adipocytes (Guo et al. 2012). Furthermore, mouse embryonic fibroblasts (MEFs) from *Bmal1*-deficient mice as well as 3T3-L1 cells with *Bmal1* knock-down fail to differentiate into adipocytes. Interestingly, when BMAL1 is overexpressed with adenoviral gene transfer, this ability is restored and cells can accumulate lipids and express adipocyte-related genes, such as *PPARγ2*. The promotor activity of these adipogenic genes is stimulated in a BMAL1-dependent manner, and the expression of adipogenic factors PPARγ2 and adipocyte fatty acid binding protein (AP2) show clear circadian rhythms in murine adipose tissue (Guo et al. 2012). Taken together, these results suggest that BMAL1 is an important factor in adipogenesis regulation.

More recently, it has been shown that *Bmal1* disruption in vivo actually leads to increased adipogenesis, adipocyte hypertrophy and obesity in global *Bmal1* KO mice (Shimba et al. 2005). Here, it has been uncovered that *Bmal1* deletion leads to down-regulation of genes in the canonical WNT signalling pathway, which are known to suppress adipogenesis. The gene promoters of several of these WNT-regulated genes, including *Wnt10a*, *β*-*catenin*, *Fzd5*, *Dvl2* and *Tcf3* displayed BMAL1 occupancy. Similarly, *Bmal1* knock down led to attenuation of WNT signalling pathway, whilst BMAL1 overexpression led to opposite effects. Stabilising β-catenin through WNT ligand administration or GSK-3β inhibition ameliorated the decreased WNT signalling and rescued inhibition of adipogenesis induced by *Bmal1* knock-down. Taken together, this study offered a mechanistic links between *Bmal1* disruption, altered adipogenesis and development of obesity in mice (Shimba et al. 2005). Another clock gene implied in adipose cell differentiation is *Rev*-*erbα (NR1D1)*, which has been shown to be a key regulator of brown adipose tissue development (Nam et al. 2015). As *Rev*-*erbα* promotes brown adipogenesis, genetic ablation of *Rev*-*erbα* impairs embryonic and neonatal brown fat formation in mice, by disrupting brown adipocyte lineage commitment and terminal differentiation. By pharmacologically activating REV-ERBα activity, brown adipocyte differentiation can be promoted, as REV-ERBα represses key components of the TGF-β cascade, which, in turn, inhibits brown fat development.

### Neurogenesis

Adult neurogenesis, which generates both new neurons and glia, is regulated by circadian rhythms. When neurosphere cultures prepared from the dentate gyrus in the brain are isolated from *mPer1::luc* clock reporter mice, it was apparent that circadian *mPer1* gene rhythms can be observed in neurospheres where neurogenesis was induced, but not in those neurospheres maintained in the stem cell state (Malik et al. 2015). In addition, neurospheres used from both *Bmal1 KO* as well as *Cry1/2* KO mice, another genetic model of circadian disruption, showed that circadian rhythms are not required for neurosphere induction in vitro. However, the absence of these clock components did restrict neurosphere growth, neuronal fate commitment and increased cell death. Quiescent neural progenitor cells (QNPs) in the subgranular zone (SGZ) of the adult hippocampus also express components of the molecular clock and proliferate in a rhythmic manner (Bouchard-Cannon et al. 2013). Here, the clock proteins PER2 and BMAL1 are essential for the control of neurogenesis. The circadian clock is crucial in timing the entry and exit of the QNPs into the cell cycle and, without these clock components, the quiescent state achieved during neuronal differentiation is delayed.

Moreover, *mPer2* is also expressed by neural stem/progenitor cells (NPCs) differentiating to mature neurons in the dentate gyrus. It has been postulated that *mPer2* provides a functional link by influencing the early cellular events that lead to post-mitotic granule cell production underlying adult hippocampal neurogenesis (Borgs et al. 2009). In the lateral subventricular zone (SVZ), the area in the brain where NPCs persist and postnatal neurogenesis occurs, the expression pattern of clock genes changes following the onset of differentiation, and *Bmal1* begins to oscillate endogenously. If *Clock* or *Bmal1* were silenced using RNA interference, the percentage of neuronal marker Map2-positive cells decreased as well as the expression level of neurogenic transcription factors such as NeuroD1 (Kimiwada et al. 2009). A study recently published by Akle et al. (2017) showed that all neurogenic niches studied in an adult diurnal vertebrate, the zebrafish, including the dorsal telencephalon, habenula, preoptic area, hypothalamus and cerebellum, showed circadian modulation of cell cycle progression that involved the use of both niche-specific and systemic factors (Akle et al. 2017).

### Osteogenesis

The circadian clock has also been implicated in bone homeostasis. *Bmal1 KO* mice display low bone mass phenotype which worsens over lifespan. These mice have decreases in cortical and trabecular bone volume and a lower bone mineral density when visualised using micro-computed tomography (Samsa et al. 2016). *Bmal1* deficiency in vivo has been shown to result in a decreased number of active osteocytes and osteoblasts, and isolated BM-MSCs displayed a reduced osteoblastic differentiation capacity, likely contributing to the observed reduction in osteoblast and osteocyte numbers (Samsa et al. 2016). Another clock gene implicated in BM-MSC proliferation and osteogenesis is *Rev*-*erbα*. It has been demonstrated that *Rev*-*erbα* expression decreases during osteogenesis. *Rev*-*erbα* overexpression led to inhibition of BM-MSC proliferation and osteogenic differentiation, which were partially rescued by activating Wnt/β-catenin signalling via exogenous Wnt3a ligand administration (He et al. 2014). This suggests that increased *Rev*-*erbα* levels could promote BM-MSC ageing and negatively regulate osteogenesis with age, which warrants further investigation.

### Chrondrogenesis

It has also been documented that *Bmal1* expression is decreased in human cartilage from osteoarthritic patients and aged mouse cartilage (Dudek et al. 2016). By ablating *Bmal1* expression in mouse chondrocytes specifically, this led to progressive degeneration of articular cartilage, most likely as a result of a number of altered molecular pathways that *Bmal1* targets. Indeed, *Bmal1* ablation in cartilage led to altered TGFβ pathway associated with an altered ratio in levels of phosphorylated SMAD2/3 versus phosphorylated SMAD1/5. Moreover, altered NFATc2 transcription factor pathway was observed, together with reduced expression of matrix related genes *Sox9*, *Acan*, and *Col2a1,* linking the circadian clock to the maintenance and repair of cartilage.

### Myogenesis

Important role for *Bmal1* has been demonstrated regarding myogenesis. *Bmal1* Is highly expressed in skeletal muscle and is thought to regulate myogenic differentiation via direct transcriptional activation of canonical Wnt signalling pathway components (Chatterjee et al. 2013, 2015). Moreover, it has been shown that a master regulator of myogenesis, MyoD, displays cyclical mRNA and protein levels, and is a direct target of CLOCK and BMAL1. *Bmal1* KO and *Clock*^Δ19^ mouse models show reductions in the total muscle mass and maximal force including reduced mitochondrial volume, demonstrating the importance of CLOCK and BMAL1 in skeletal muscle structure and function (Andrews et al. 2010). Similarly, when *Bmal1* is knocked down in myoblasts, this led to impaired myogenic differentiation and decreased expression of key myogenic regulatory factors including *Myf5*, *Mrf4* and *Myogenin* as well as *Myosin Heavy Chain 3 (MHC3)*. Overexpression of *Bmal1* in C2C12 myoblasts conversely led to accelerated myogenesis and attenuation of Wnt signalling, indicating that *Bmal1* is required for myoblast differentiation (Chatterjee et al. 2013) Direct binding of BMAL1 to gene promoters coding for canonical Wnt pathway genes has been demonstrated in line with circadian oscillations of several Wnt pathway components (Chatterjee et al. 2015). *Bmal1* can also influence skeletal muscle function through influencing its regeneration process (Sun et al. 2014). *Bmal1 KO* mice display significantly lower satellite cell expansion, which leads to defective regenerative responses. These mice exhibit a nearly non-existent induction of Pax7, a satellite cell marker. The satellite cell-derived myoblasts isolated from *Bmal1 KO* mice demonstrate reduced growth and proliferation ex vivo, underscoring the role of *Bmal1* in both muscle maintenance and repair (Sun et al. 2014).

### Angiogenesis

Circadian clock protein PER2 is thought to be a key factor in maintaining endothelial progenitor cell (EPL) function during angiogenesis. PER2 is abundantly expressed in early EPCs, whilst EPCs from *Per2*^−*/*−^ mice demonstrate impaired proliferation, adhesion, migration and tube formation due to inhibitions of both PI3K/Akt/FoxO signalling and protein levels of C-X-C chemokine receptor type 4 (CXCR4). Interestingly, the negative proliferation effects of *Per2* deficiency can be rescued by activating PI3K/Akt/FoxO signalling. Indeed, PER2 and CXCR4 directly interact in EPCs, showing that PER2 is essential in maintaining early EPC function (Al Mheid et al. 2014). In humans, circulating progenitor cells and their proangiogenic activity also exhibit circadian variations. They show unfavourable profiles in the morning which coincides with prevalence of cardiovascular events, directly influenced by the endogenous circadian clock (Bhatwadekar et al. 2017). When *Bmal1* is conditionally deleted in endothelium and hematopoietic cells specifically, cellular responses to microvascular and macrovascular injuries are exaggerated (Dierickx et al. 2017), highlighting the importance of circadian rhythms in maintaining vascular homeostasis.

### Other tissue-specific cell differentiation

In the cardiac muscle, circadian rhythms regulate cardiac physiology and metabolism and are known to determine outcomes of ischemic stress (Van Laake et al. 2018). Human ESCs progressively oscillate following directed cardiac differentiation. Furthermore, a number of clock-controlled output genes in the heart have been identified as an oscillatory network of stress-related genes, including genes known to play an important role in human heart physiology, including *Pln*, *Kcne4*, *Tspo*, *Cav1* and *Rgs2* (Dierickx et al. 2017). Stem cell antigen 1-positive (SCA1+) cells, which can be detected in the heart, have been shown to possess a molecular clock and exhibit circadian oscillations that control downstream cellular functions (Du Pré et al. 2017). These findings demonstrate the importance of circadian regulation in cardiovascular functions.

**Fig. 3:**
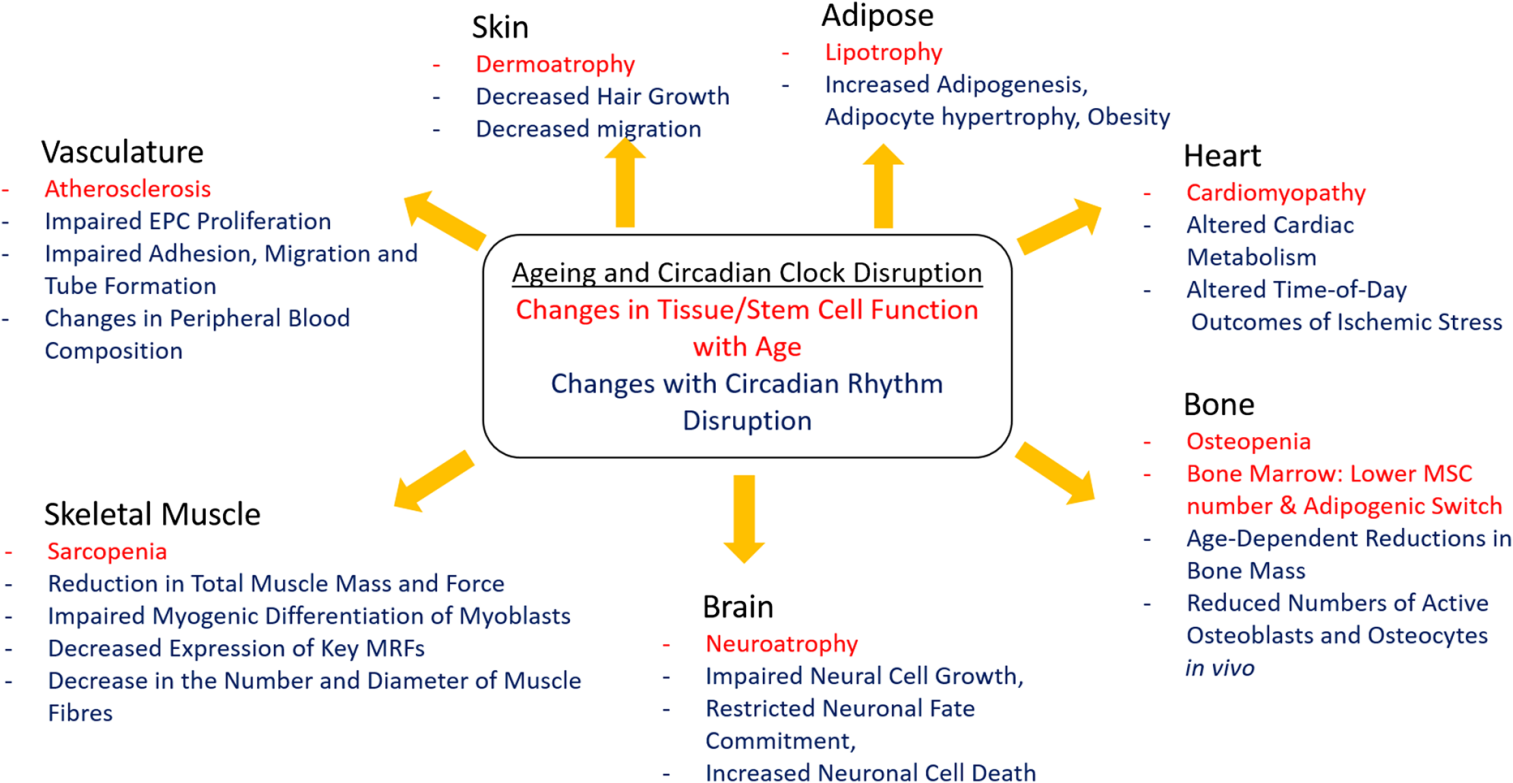
Summary of the Common Changes seen with Ageing and Circadian Clock Disruption

Intestinal stem cells (ISCs) are critical in determining how the intestine regenerates in order to replace dying cells. The PER transcription factor has been discovered to be essential in intestinal regeneration, and numerous gene transcripts that are regulated by the circadian clock have been uncovered within intestinal stem cells, including those involved in stress response and regeneration pathways (Karpowicz et al. 2013). Disruption of clock component *Per* has been shown to lead to arrhythmic ISC divisions, demonstrating how diverse roles in stem cells are played by the components of the molecular clock in different peripheral tissues (Karpowicz et al. 2013). A summary of tissue phenotypes associated with disrupted circadian rhythms which are commonly found in various tissue systems affected during ageing is shown in Fig. 3.

## The impact of the circadian rhythms on stem cell homeostasis and cell cycle progression

Research by Boucher et al. (2016) has demonstrated the effects of the clock genes on MSC differentiation, migration and cell cycle regulation. They showed that knock down of *Clock* or *Per2* led to inhibition of adipocyte differentiation, while osteoblastic differentiation was unaffected. Cell migration was decreased in *Per2* KD cells together with altered hMSC cell cycle stage distribution. This was in line with observed changes in cyclin expression profiles including significant decreases in several cell cycle regulators p19, p27, Cyclin B1 and Cyclin D1 protein levels (Boucher et al. 2016). This is further evidence that the circadian clock in stem cells is important in maintaining their function and properties.

Further research examined the role of the clock in cell cycle regulation of hair follicle stem cells (HFSCs) that reside within the bulge (Zagni et al. 2017). One of the major regulators of the PI3K/AKT pathway, PTEN, has a critical role in controlling HFSC number and size, and maintaining the pluripotent state of stem cells. PTEN and BMAL1 show functional links; when *Pten* is deregulated, this causes constitutive activation of BMAL1, and BMAL1 is involved in the maintenance of the PTEN-induced phenotype. Short-term and long-term *Pten* depletion leads to BMAL1 activation, which contributes to HFSC accumulation.

Another important aspect of circadian clock regulation of stem cells relates to the ‘clock-gated’ cell division cycles. Using 3D murine organoids, researchers have demonstrated Wnt-mediated intercellular coupling between the cell cycle and the circadian clock (Matsu-ura et al. 2016). The molecular clock ‘gates’ the existence of cell populations with heterogeneous cell cycle times generating self-synchronised, 12-h cell division cycles. It is thought that an intercellular signal linking circadian and cell division cycles is established by differentiated cells (Matsu-ura et al. 2016). A summary of the signalling pathways that affect stem cell activation, proliferation and differentiation, which are under circadian control, can be found in Table 2.

**Table 2:** Signalling Pathways Controlled by the Circadian Clock relevant to Adult Stem Cell Function

Signalling pathways	Role in adult stem cells	References
WNT	Differentiation	Janich et al. (2011), Guo et al. (2012), Chatterjee et al. (2013), Chatterjee et al. (2015)
NOTCH	Self-renewal	Zhang et al. (2017) and Janich et al. (2011) Several factors bound by *Bmal1*
p53	Apoptosis	Yagita et al. (2010) Activated by *Per2*
Cyclin B	Cell cycle	Plikus et al. (2013), Boucher et al. (2016)
PI3K/AKT	Homeostasis/proliferation	Sun et al. (2014), Zagni et al. (2017)
TGF-β	Homeostasis/proliferation	Moerman et al. (2004), Janich et al. (2011) and Plikus et al. (2013)
BMP	Proliferation	Janich et al. (2011)
Rho/ROCK ROS/NRF2	Homeostasis Homeostasis	Yang et al. (2017) Kondratov et al. (2006), Kondratov et al. (2009) and Pekovic-Vaughan et al. (2014)

Furthermore, circadian clock is implicated in several cellular protective mechanisms that are important for stem cell homeostasis such as antioxidant stress responses (Kondratov et al. 2009). Reactive oxygen species (ROS) are produced as a by-product of cellular metabolism and serve as signalling molecules. However, increased ROS production and/or clearance results in oxidative stress, leading to oxidative damage to major macromolecules including proteins, lipids and DNA, which is associated with tissue degeneration with ageing (Paul et al. 2014; Dickinson and Chang 2011). Importantly, genetic disruption of *Bmal1* in mice leads to altered redox homeostasis with increased accumulation of ROS in several tissues (Kondratov et al. 2006), which can be partially ameliorated with administration of glutathione precursor *N*-acetyl cysteine (NAC) (Kondratov et al. 2009). Moreover, *Clock*^Δ19^ mice show diminished levels of antioxidant transcription factor *Nrf2* and its target antioxidant genes leading to an increased accumulation of protein oxidative damage in the lungs with ageing (Pekovic-Vaughan et al. 2014). CLOCK and BMAL1 heterodimer were found to positively regulate *Nrf2* transcription by temporal control of the E-box element in the *Nrf2* gene promoter. NRF2 protein, in turn, drives the rhythmic transcription of antioxidant genes via temporal binding to antioxidant response elements (AREs) in several gene promoters encoding enzymes involved in glutathione synthesis and utilisation. The lack of NRF2-mediated antioxidant defence provides a potential mechanism for the premature onset of age-related pathologies seen in the absence of *Bmal1* in mice (Pekovic-Vaughan et al. 2014).

## The effects of circadian rhythms on stem cell biomaterials and tissue engineering

In regenerative medicine, there is a great need to generate mature functional tissues in vitro, especially given our increasingly ageing society. Therefore, it is essential to discover effective means to direct differentiation of stem cells into tissue-specific cells in a controlled manner, which requires appropriate biochemical and biophysical signals within cellular microenvironments. Biomaterials provide a 3D environment equipped with biological, mechanical and chemical cues, which can stimulate cells to proliferate, differentiate, secrete extracellular matrix and form functional tissues (Hwang et al. 2008; Burdick and Vunjak-Novakovic 2008; Singh and Elisseeff 2010; Lutolf et al. 2009; Dawson et al. 2008). Intelligent biomaterials that can mimic the required physical and biochemical environments and provide the necessary signals for stem cell activation and differentiation have recently undergone intense research. Many types of biomaterials have been developed, including the use of natural materials, which consist of extracellular matrix components such as collagen, fibrinogen, hyaluronic acid, glycosaminoglycans (GAGs), laminin and hydroxyapatite (HA), as well as synthetic materials, such as polymers, ceramics and metals. There has also been a drive to utilise surface modifications of biomaterials, including chemical and biological modifications, so that the differentiation of stem cells may be selectively steered down particular cellular fates. Several articles have reviewed in detail the application of biomaterials in stem cell differentiation (Dawson et al. 2008; Hwang et al. 2008; Burdick and Vunjak-Novakovic 2008; Singh and Elisseeff 2010; Lutolf et al. 2009).

The circadian rhythms have recently been implied in dental and orthopedic implant integration. In order to investigate causes of failure in osseo-integration, an implant failure model under vitamin D deficiency was utilised to identify crucial gene networks that underpin this process (Mengatto et al. 2011). Following genome-wide transcriptomic and bioinformatic molecular pathway analyses, it was reported that the circadian rhythm pathway was among the top molecular pathway affected. *Npas2* and *Bmal1* were upregulated around the implant and diminished by vitamin D deficiency, whereas the *Per2* expression pattern showed the opposite trend. It was thus concluded that the circadian rhythms, along with the extracellular matrix, may be involved in osseo-integration establishment under vitamin D regulation.

In a recent follow up study by Hassan et al. (2017), the circadian rhythm of BM-MSCs was induced using titanium-based biomaterials with complex surface modifications (Hassan et al. 2017). When cultured on such materials, BM-MSCs supressed *PER1* expression and upregulated *NPAS2*. When further investigated using *Npas2* KO mice, the titanium biomaterial-induced suppression of *Per1* was not rescued and the expression of other clock genes *Per2, Per3, Bmal1* and *Clock* was not affected, suggesting that the altered expression of *Per1* was independent of *Npas2*. The authors concluded that titanium-based biomaterials can influence BM-MSC circadian rhythms, and altered BM-MSC rhythms may be important factor in determining the rate of titanium-based biomaterial integration into bone. This exciting area of research examining how materials influence the stem cell clock warrants further research, and the authors of this review are optimistic that it will be a very prosperous area of research to come.

The circadian clock has recently been implicated in regenerative medicine as it has been shown to have a profound effect on the wound healing response in mice and humans (Hoyle et al. 2017). Skin wounds in mice healed faster in the active period than those incurred in the rest period ex vivo and in vivo. Using a proteome-wide screen for rhythmically expressed proteins in fibroblast cells, the authors uncovered a circadian regulation of actin, a cytoskeletal protein involved in cell migration, which is an important aspect in the wound-healing response of fibroblasts and keratinocytes (Hoyle et al. 2017). Analysis of a database of human burn injuries showed that human burns patients who incurred their injuries in the night, i.e. rest period, healed more slowly than those incurred in the day, i.e. active period. This research highlights the importance of the circadian regulation of the cellular cytoskeleton in modulating wound healing responses and underlines the importance of the circadian rhythms in regenerative medicine.

## The effects of the rhythmic mechanical cues on stem cell clocks

The circadian clock has a period of approximately, but not exactly, 24 h. Therefore, it must be reset daily by external cues, known as *Zeitgebers*. The most common of these cues is light which, in mammals, entrains rhythms in the SCN through retino-hypothalamic tract. The SCN relays this temporal information to the rest of the brain and peripheral tissue clocks via diffusible signals and neuroendocrine factors (Silver et al. 1996). As the mammalian core body temperature itself oscillates in a circadian manner, this too can act as a *Zeitgeber*, as was initially shown by subjecting cultured fibroblasts to rhythmic temperature oscillations (Brown et al. 2002). This temperature entrainment was demonstrated to be sufficient to sustain circadian rhythmicity in vivo, and abnormal temperatures cycles were reported to cause decoupling of peripheral oscillators from the SCN (Brown et al. 2002). Glucocorticoids, which are a class of steroid hormones that bind to the glucocorticoid receptor (GR) present on almost every vertebrate cell surface (except the SCN), have also been implicated in synchronising peripheral circadian rhythms in human and murine ASCs (Balsalobre et al. 2000; Wu et al. 2008). Huang et al. (2009) have demonstrated that human stem cells have circadian oscillations that can be induced by serum shock and cAMP analogues in vitro (Huang et al. 2009), showing that stem cells can be synchronised using hormones and growth factors.

One emerging entrainment mechanism of circadian clocks is mechanical stimulation. Indeed, in the body, different cells in various anatomical locations are subjected to varying amounts of mechanical strain. Published data has demonstrated that a uniaxial strain between 5 and 15% with a frequency of 1 Hz has notable effects on MSCs both on proliferation and collagen synthesis (Sun et al. 2016). Furthermore, O’Caerbhaill et al. (2008) highlighted that radial distensions of 5% and frequencies of 1 Hz caused mechanosensitive effects on stem cells including cell reorientation parallel with direction of flow and altered cellular morphologies, highlighting that there is a significant cytoskeletal restructuring in mechanically stimulated MSCs compared to static cells.

Recent research has also demonstrated that mechanical vibrations have the capability of resetting the clock in *Drosophila melanogaster*. It has been shown that rhythmic mechanical stimulation of the chordotonal organs can synchronize the *Drosophila* circadian clock (Simoni et al. 2014). Loss-of-function mutation in the *Period* gene led to impaired ability of mechanical synchronization, highlighting the importance of a functional clock system for mechanical entrainment. Research from our own group has recently demonstrated that cyclical mechanical stretch can be used to synchronise circadian rhythms of human ASCs (Rogers et al. 2017). Here, primary human ASCs from bone marrow, dental pulp and adipose tissues were synchronised using a novel mechanical cell stretch paradigm (12 h ON: 12 h OFF cyclical uniaxial stretch for three days) and the expression of core clock genes analysed over two circadian cycles in the absence of rhythmic stimulation. Rhythmic mechanical stimulation was sufficient to synchronise circadian rhythms in distinct ASCs, with differential propensity for mechanical synchronisation displayed by ASCs derived from distinct human tissue locations. Interestingly, mechanical strain has also been shown to inhibit adipogenesis in MSCs by stimulating a robust β-catenin signal (Sen et al. 2008), which is in line with the previous reports showing that *Bmal1* influences adipogenesis through β-catenin pathway. Whether BMAL1 has a role in regulating mechano-transduction pathways to influence adipogenesis is currently unknown and warrants further investigation. A summary of the circadian synchronising mechanisms that have been investigated in ASCs is presented in Table 1.

A recent study has demonstrated the importance of cell–matrix interactions for stem cell clocks and discovered that the mammary epithelial clock is regulated by the mechanical stiffness of the cellular microenvironment (Yang et al. 2017). The authors demonstrated that genetic disruption of clocks compromises the self-renewal ability of the mammary epithelial stem cells, underlining the key link between clock genes and mammary stem cell function. Interestingly, the authors demonstrated a functional link between tissue matrix stiffness with age and the amplitude changes in clock oscillations. Increased tissue stiffening with age was shown to suppress the amplitude of the mammary clock oscillations through the tension sensing cell–matrix adhesion molecule, vinculin, and the Rho/ROCK signalling pathway. This mechano-transduction signalling pathway is, in turn, transduced into the cell to regulate the activity of core clock machinery. In this study, the authors also investigated whether the circadian rhythms in mammary tissues isolated from old mice can be restored by administering drugs to alter matrix stiffness. ROCK pathway inhibitors were used, implicated in matrix stiffness regulation, which led to improved amplitude of clock gene oscillations in mammary tissues from old mice (Yang et al. 2017). Therefore, it was concluded that tissue stiffening seen with ageing is thought to suppress the mammary clock in vivo, providing a mechanism of how ageing disturbs the mammary epithelial stem cell clock through altering the stem cell niche.

## Summary

The field of circadian stem cell biology is a dynamic area, which has provided us critical insights into the temporal control of stem cell function and maintenance. Whilst these are exciting developments, there is much fundamental research that is still needed as our understanding in this crucial area continues. Key aspects to build on over the next few years include the bi-directional effects of the biomaterials and biomechanical factors on stem cell clocks. Future research assessing the role of epigenetic factors on stem cell clocks, which are well documented to influence both circadian rhythms and ASC function, will be of great importance to the application of circadian biology to stem cell research. In summary, the invaluable knowledge pertaining to temporal regulation of stem cell physiology and metabolism by tissue-specific circadian clocks will provide novel insights into the dynamic processes that change during stem cell ageing, and allow appropriate optimisation of smart biomaterials and mechanical cues essential for steering stem cells into particular cell fates. Such critical findings are essential for future design of novel cellular therapies to ameliorate and/or slow down a number of age-related diseases and begin to tackle their prevention.
